# Experience-based co-design of an active case finding service for colorectal cancer in community pharmacies: findings from a focused ethnography

**DOI:** 10.1186/s40900-025-00740-0

**Published:** 2025-06-10

**Authors:** Naseeb Ezaydi, Daniel Hind, Atique Arif, Ian Kellar, Rachel Matthews, Dana Marbu, Claire Thomas, Matthew Kurien, DETECT-CRC Co-design group, Louise Merriman, Akeela Mohammed, Salma Hussain, Siama Akhtar, Jackie Hatton, Shirley Samuels, Erum Rehman, Shabir Aziz, Abdul Rehman Khalid, Tasleem Aziz, Shirley Kirkland, Carole Hobson, Steven Dexter, Peter Hewkin, Michelle Ord, Yvonne Witter, Stephanie Edgar, Thomas Bisset, Richard Hackett, Ellie Bennett, Rosemary Blackie, Tina Cooke

**Affiliations:** 1 School of Medicine and Population Health, Sheffield Centre for Health and Related Research, University of Sheffield, Sheffield, UK; 2 Senior Research Fellow (Social Care Research and Development), School of Healthcare, University of Leeds, Leeds, UK; 3 Rotherham Doncaster and South Humber NHS Foundation Trust (RDaSH), Doncaster, UK; 4 Department of Psychology, The University of Sheffield, Sheffield, UK; 5 Freelance Certified Professional Co-Active Coach and National Voices Associate, Rachel Matthews Coaching and Consultancy, London, UK; 6 Community Pharmacy South Yorkshire, Nuneaton, UK; 7 Division of Clinical Medicine, School of Medicine and Population Health, The University of Sheffield, Sheffield, UK

**Keywords:** Patient and public involvement, Co-design, Early diagnosis, Cancer detection, Pharmacy

## Abstract

**Background:**

Colorectal cancer (CRC) is the fourth most common cancer and second leading cause of cancer deaths in the UK. Socioeconomic deprivation is associated with delayed diagnoses and poorer CRC outcomes. Community pharmacies, highly accessible in underserved areas, present an opportunity to address these health inequalities. This DETECT-CRC study aimed to develop a pharmacy-based active case-finding (ACF) service for CRC in underserved communities of Yorkshire, UK.

**Methods:**

We used a modified Experience-Based Co-Design (EBCD) approach to develop the ACF service. Four co-design workshops were conducted over five months, bringing together pharmacists, general practitioners (GP), patients and community members. A focused ethnography was embedded within the EBCD process, consisting of interviews and observation of workshops. Field notes were analysed thematically to identify key considerations shaping the service design.

**Results:**

Three overarching themes emerged: 1) Amplifying community pharmacy assets, emphasising accessibility and trust-building; 2) Strengthening inclusive practice, highlighting privacy, cultural considerations, health literacy, and emotional factors; and 3) Enabling service integration and quality, stressing collaboration between pharmacies and GPs, and pharmacy training needs. These insights informed the development of a comprehensive ACF service model, including multilingual patient-facing materials, a training package for pharmacy staff, and protocols for GP communication. The co-design process ensured the resulting service was grounded in community needs and perspectives.

**Conclusion:**

This study provides a co-designed model for pharmacy-based ACF of CRC in underserved areas. The model shows promise in addressing health inequalities and improving early cancer detection. It demonstrates how community pharmacies can play a pivotal role in cancer detection, contributing to the NHS Long Term Plan’s ambition of diagnosing 75% of cancers at stage 1 or 2 by 2028. While further research is needed to evaluate its effectiveness, this approach holds potential for improving CRC outcomes in underserved communities and could be adapted for other health conditions and settings.

**Supplementary Information:**

The online version contains supplementary material available at 10.1186/s40900-025-00740-0.

## Abstract (plain language summary)

Colorectal cancer is the fourth most common cancer in the UK and the second leading cause of cancer deaths. People living in poorer areas get screened for this cancer less often. This means their cancers are usually found at later, less treatable stages. The DETECT-CRC project aimed to make it easier for people to get tested by involving local pharmacies. Nearly everyone lives close to a pharmacy, and most people visit one at least once a year.The research team worked together with members of underserved communities, doctors, and pharmacists in a series of workshops to design a new pharmacy service, where people who have potential symptoms of colorectal cancer can get tested using a simple home test. During these workshops, people talked about what might help or get in the way of using a new pharmacy service, and shared their experiences with other pharmacy services. The main points people talked about in the workshops were:

• Making the most of what community pharmacies can offer.

• Making sure the service works well for everyone.

• Fitting the service in with other health services.

From the workshops, a new pharmacy service was designed with local people and professionals. The new service uses local pharmacies, which are easy to get to and trusted by many people, to help find signs of bowel cancer earlier in areas where healthcare is harder to access. We think this new approach could help catch cancer earlier in these communities, which might help reduce unfair differences in health care.

**Supplementary Information:**

The online version contains supplementary material available at 10.1186/s40900-025-00740-0.

## Background

Colorectal cancer (CRC) is a significant health challenge in the United Kingdom, representing the fourth most common cancer and the second leading cause of cancer-related deaths. Early diagnosis is crucial for improving CRC outcomes, with a notable difference in five-year survival rates between stage 1 (95%) and stage 4 (10%) diagnoses [1, 2]. In England, general practitioners (GPs) invite all people aged 60 to 74 to take part in a bowel cancer screening programme every two years, using a Faecal Immunochemical Test (FIT). This is called ‘population screening’ and, whilst it improves the detection rate overall, can increase health inequalities due to lower screening uptake in low socioeconomic groups [3].

In Yorkshire, CRC accounts for over 3,500 new diagnoses annually, representing over 10% of the total diagnoses in England [4, 5]. The region faces particular challenges, with around 20% of Yorkshire and the Humber’s population living in areas classified within the lowest decile of the Index of Multiple Deprivation, and lower life expectancy compared to the national average [6]. This socio-economic deprivation directly correlates with poorer CRC outcomes, including delayed diagnoses and more advanced stages at presentation [7, 8]. These regional inequalities highlight an urgent need for targeted interventions in Yorkshire’s most vulnerable communities.

While health inequalities in oncology are well-documented, a recent scoping review called for a shift from merely identifying these disparities to actively addressing them [9]. The DETECT-CRC study responds directly to this call by testing innovative approaches to improve early CRC detection in underserved populations. Specifically, we aimed to assess active case finding (ACF) of CRC through community pharmacies in socio-economically deprived areas of Yorkshire, UK. ACF is distinct from population screening, which largely targets people without symptoms. ACF is a systematic approach which seeks to identify individuals who may not recognise their symptoms or face barriers in accessing healthcare, particularly in the communities most affected by disparities in cancer outcomes [10]. Individuals from underserved communities feel they are treated differently by primary care providers with anxiety around discussing sensitive health topics identified as a barrier to accessing services [11]. A number of studies in the past five years have shown that people frequently present with alarm symptoms for cancer at community pharmacies, that screening and testing by pharmacies can increase early cancer detection, but that further research is needed to develop formal referral pathways, integrating community pharmacies with general practice, with adequate training, workflows and reimbursement [12–15].

Community pharmacies are accessible due to their widespread geographic distribution, with 89.2% of the English population living within a 20-min walk of a pharmacy [16]. This accessibility is particularly high in underserved areas, positioning pharmacies as a unique resource for engaging these communities [16]. Pharmacies function as accessible health-assessment venues and can provide a range of health-promoting services beyond medication dispensing [17, 18]. Systematic reviews have suggested that community pharmacies could significantly contribute to cancer education and screening [17, 19], with several studies demonstrating the feasibility and potential of pharmacy-based CRC interventions [20–22]. However, further research is needed to optimise the delivery of such interventions, demonstrate their effectiveness and underpin them with robust theory [19].

The DETECT-CRC study addresses this gap by developing a pharmacy-based ACF service for CRC in underserved communities, in which individuals with suspected CRC are offered faecal immunochemical tests (FIT) through community pharmacies. The project invited key stakeholders (including pharmacists, general practitioners, patients, community members) to co-design the ACF service, aiming to improve its acceptability and feasibility. Service co-design followed a modified Experience-Based Co-Design (EBCD) approach, engaging key stakeholders to refine the ACF service models by using their lived experience.

In this paper we report a focused ethnography that documented the development of the pharmacy-based ACF service. The ethnographic study was embedded within the EBCD process where stakeholders came together to share views and experiences, identify uncertainties, and inform the service design.

Focused ethnography is a pragmatic, time-limited adaptation of traditional ethnography, characterised by short-term field visits, a focus on a discrete community or activity, and intensive data collection and analysis [23]. It was well-suited for studying specific aspects of contemporary society and informing interventions, making it appropriate for this service design context. By observing the co-design process, the ethnography aimed to capture the collaborative sense-making and negotiation that underlay the final service design, providing insights that could enhance the service’s implementation and transferability.

### Aim and objective

The overall aim of the DETECT-CRC programme was to assess the feasibility of active case-finding for colorectal cancer in socio-economically deprived areas using community pharmacies, aiming to improve the effectiveness and cost-effectiveness of case-detection. This supports the NHS England target of detecting 75% of cancers at Stages 1 or 2 by 2028 [24–26]. This aim also aligns with the NHS England approach on addressing inequalities by focusing on the most deprived 20% of the national population [25]. Therefore, it aligns with national and regional priorities in cancer care and pharmacy practice [26].

The research question for our programme of work is: “can active case-finding of CRC in pharmacies be effectively used within underserved communities?”.

The specific objective of the work described in this paper was to co-design a pharmacy-based ACF service model for colorectal cancer detection in underserved areas. The service will aim to integrate community pharmacies into the colorectal cancer detection pathway, leveraging the accessibility and trust of pharmacies to engage individuals with potential symptoms, offer them FIT kits through private consultations, and promptly communicate results to patients and their GPs for follow-up.

To deliver on this objective we employed a modified Experience-Based Co-Design (EBCD) approach, engaging pharmacists, GPs, patients, and community members, in a series of co-design workshops to collaboratively design the ACF service. A focused ethnography was embedded within the EBCD process to document the collaborative sense-making and negotiation underlying the final service design. The feasibility and effectiveness of the co-designed ACF service will be tested in a cluster randomised controlled trial (ISRCTN: 14,156,362) and reported separately. The reporting of this work has been completed in line with the Guidance for Reporting Involvement of Patients and the Public (GRIPP2) checklist (see Additional file 1) [27].

## Methods

### Research team and reflexivity

In line with best practice and to enable effective triangulation, our research team included members with diverse perspectives and expertise, including ‘outsider’ academics and ‘insider’ community and professional members, who played a key role in building trust with participants, contextualizing findings, and facilitating organisational responsiveness to the research [28]. Data collection and analysis for the focused ethnography were led by a graduate anthropologist (DH) and an experienced applied health researcher (NE). All authors participated in organising the coproduction (led by MK, RM, IK) and brought various experiences, including: group facilitation (RM, IK); co-producing services (IK, RM, DH, NE); quality improvement (RM, DH, NE); implementation science (IK, DH); identifying as non-white (DM, NE, MK, AA); being a medic involved in early detection of bowel cancer (MK); being a community volunteer worker in underserved areas and a research ambassador (AA); being a community pharmacist (CT). Team diversity is critical in establishing credibility with community members [28]. In terms of values, we were united by the conviction that service changes, as proposed by NHS England’s model for pharmacy-based detection, are not feasible or acceptable unless co-designed by relevant stakeholders [29]. We are all committed to reducing health inequalities and improving care access, experience, and outcomes for underserved populations. More broadly, this work is a response to the Inverse Care Law and the Marmot Review, recognising that: “disadvantaged populations need more health care than advantaged populations, but receive less” [30, 31]; and, social determinants significantly impact health outcomes and engaging underserved communities in co-creation of accessible healthcare services is necessary to address the social gradient in unequal health outcomes [32, 33].

### Experience Based Co-Design (EBCD)

EBCD’s philosophical commitments mirror those of community-based participatory research in public health, particularly equitable involvement of end-users and professionals, experience-led inquiry and joint meaning-making and practical action grounded in lived experience. EBCD’s originators explicitly draw on participatory approaches in terms of system design [34], the priority of the service user [35, 36], and the need to make emotional connections [37, 38]. Advocates also draw deeper connections to participatory approaches which advocate collaborative problem investigation, reflective inquiry, action for practical, shared benefit and partnerships between researchers and participants throughout the process [39–41]. EBCD has been shown to enhance intervention development by aligning patient and staff perspectives [42, 43]. This method brings together staff and service users to collaboratively design and improve services by using their lived experience [43]. The standard format follows six key stages: (1) gathering experiences; (2) identifying key touchpoints; (3) developing and using trigger films; (4) conducting patient and staff feedback sessions; (5) co-design workshops to prioritise improvements; and, (6) implementing and sustaining changes [44]. We drew on previous work that utilised EBCD methodology across patient pathways to adapt the EBCD process [45]. We followed a modified EBCD approach to refine the service model [44]. The modifications arose because: (a) We were refining an existing service, and heavily constrained by existing guidance, not building a service from the ground up; and, (b) we had been advised that the stigmatised nature of the symptoms and mistrust in research in underserved communities precluded some elements. Instead of conducting formal, filmed narrative-based interviews, we carried out a series of interviews with key stakeholders. These interviews informed the co-creation of personas, which were then refined in user journey mapping exercises during the workshops. This approach allowed us to capture diverse perspectives while respecting the sensitivity of the subject matter and the potential discomfort community members might feel discussing their symptoms on camera. Our persona development process involved collecting stakeholder interview data, synthesising common experiences, creating initial persona drafts, and refining them through workshop exercises. This adaptation preserved EBCD’s patient-centered focus while addressing privacy concerns and cultural sensitivities—an approach successfully used in previous studies [46].

Stage 1: We arranged semi-structured interviews with key stakeholders to build relationships and extract stimulus material for the planned co-design workshops. We conducted online interviews with one patient, six GPs and six pharmacists from across South Yorkshire. Our topic guide can be found in Additional File 2. We also gathered community experiences through two patient and public involvement community group meetings in Doncaster and Rotherham. In accordance with our research ethics committee-approved protocol, these conversations were not audio recorded or transcribed, as they were conceptualised as service development activities rather than formal research data collection. Discussions were focused on the anticipated barriers and facilitators for an ACF service, along with their experiences involving pharmacy-delivered services. Researchers took field notes during these discussions, which were used to abstract stimuli (discussion points) for the subsequent co-design workshops in Stage 3. Rapid qualitative analysis was conducted on interview content to identify the key mechanisms of action that were relevant to accessing a pharmacy service, such as knowledge, beliefs about capabilities, and environmental context and resources [47]. These mechanisms of action were then used to create initial draft personas that embodied the typical barriers, motivations, and experiences reported by the interviewees. These personas were developed during the workshops, where participants could provide additional insights and ensure the personas accurately represented the target population’s experiences and challenges.

Stage 2: We brought together members of underserved communities, pharmacists, GPs, gastroenterologists, and information specialists in an initial online co-design event led by an independent facilitator. This was attended by thirteen participants (two GPs, one information specialist, six pharmacists, three people from different communities in the proposed study area, and one person with lived experience of CRC).The purpose of the meeting was to introduce the project and the aim of the co-design work. The principles by which we planned to conduct the co-design work were outlined including acknowledging power and privilege, and embracing disagreements. In line with best practice [28], we deliberately invited participants we knew to be dissatisfied by the status quo or sceptical of the proposed service. Attendees were also invited to share what motivated them to be involved in the project, and any initial considerations regarding the proposed ACF service.

Stage 3: We held three in-person co-design workshops with community members, GPs, pharmacists and patients over five months. Attendees included those who were consulted during stage 1 and members of South Yorkshire place-based patient and public involvement groups, of which many were in underserved neighbourhoods. A breakdown of the participants attending each workshop can be found in Additional File 3. We also held an online co-design workshop for pharmacists to discuss and address training needs for the ACF service.

The workshop methodology incorporated a structured framework of shared rituals and conventions, designed to foster inclusivity and disrupt established power dynamics. These elements were consistently implemented across all workshops and included: clear communication of expectations prior to the event, a formal welcome procedure, participant introductions, overview of the workshop’s objectives and structure, establishment of collaborative working norms and a defined conclusion process. The questions and prompts used to guide discussions in each of the workshops are shown in Additional File 4.

The co-design workshops were led by a Certified Professional Co-Active Coach with extensive experience in healthcare and patient engagement. The facilitation approach was guided by principles of “Do with” and “Decide together,” established collaboratively with participants at the first meeting and reaffirmed at each subsequent workshop. To promote inclusive participation, the facilitator employed specific strategies: participants introduced themselves based on their desired contribution rather than professional titles, disrupting traditional power hierarchies; the Co-Active Coaching Model was applied to hold each participant as “naturally creative, resourceful and whole”; and differing perspectives were explicitly framed as valuable data to approach with curiosity. Conflict management was integrated through normalising disagreement and using situational awareness to surface tensions for group discussion. The facilitator demonstrated adaptability when responding to unexpected challenges, including community incidents affecting participants and personal bereavements, making appropriate accommodations while maintaining progress toward workshop objectives. This balanced approach ensured both psychological safety and productive outcomes, with time and task boundaries maintained through transparent re-contracting when necessary.

The co-design workshops brought together pharmacists, GPs, patients and residents of underserved communities in South Yorkshire, UK. Each workshop (lasting 2 h) was facilitated by an independent chair from National Voices (RM), a coalition of health and social care charities. In-person workshops were held in April, June, July and September 2024 at a venue suggested by a number of attendees in the planning stages as ‘neutral, ‘non-institutional’ and easily accessible from across South Yorkshire. Workshops occurred during the evening on the advice of pharmacists and GPs, who could not attend during working hours. Attendees were recruited through existing relationships with key stakeholders (Stage 1), adverts in regional pharmacy newsletters and reaching out to South Yorkshire place-based patient and public involvement groups. Attendees were offered a £50 payment for their time at each workshop.

• Workshop 1: Draft patient personas were created based on the stimulus material from stage 1. The personas varied in ethnicity, sex, age and attitudes towards the healthcare system. These draft personas were critically evaluated and refined by participants, who challenged potential stereotypes (such as assumptions about language preferences) while enriching the personas with more authentic characteristics. The persona elaboration questions probed key themes including psychosocial factors (such as handling health-related stress and the influence of social support networks), health literacy regarding specific conditions and preventive care, trusted information sources, and communication preferences (including language needs). They also explored sociocultural influences, encompassing how cultural background, family, and personal experiences shape attitudes towards health, healthcare, and aging, as well as individual perspectives on aging, proactive health management, and future health aspirations.
- Attendees were then asked to examine each step of the proposed pathway, and consider the experience from the viewpoint of the personas, identifying touchpoints, emotional responses and friction points. This workshop was attended by seventeen participants (one GP, five pharmacists, one person with lived experience of CRC and ten community members from the proposed study area including refugee and asylum seekers).
• Workshop 2: The focus of this workshop was on the consultation stage of the service pathway. Attendees worked together to adapt and simplify the NICE DG56 FIT criteria [48]. These criteria are currently used in primary care for triaging at-risk individuals, making them appropriate and implementable within community pharmacy settings. They drafted pictorial representations of symptom criteria to address communication barriers. A conversation guide for pharmacists was created based on a role-play exercise in which attendees developed personas and simulated a consultation (Additional File 7). Attendees were also asked to discuss sources of mistrust in communities which could prevent individuals using the service and how to address these issues. This workshop was attended by fifteen participants (three pharmacists, one hospital service coordinator, one person with lived experience of CRC and 10 community members).
• Workshop 3: Attendees reviewed materials that were drafted based on the feedback from previous workshops; this included a FIT information sheet, pharmacist conversation guide, symptom poster, results letters and letter to GPs. We also discussed what expectations the local community would have of pharmacists delivering the service in terms of knowledge, experience and skills. This workshop was attended by fifteen participants (two GPs, one pharmacist, one person with lived experience of CRC and eleven community members).
• Pharmacy workshop: The aim of this online workshop was to explore the training needs of pharmacies. One service manager and fifteen pharmacists from seven different pharmacies attended. We began by checking assumptions on how pharmacies deliver services including roles, responsibilities, accountability and continuing professional development requirements. We then discussed what pharmacies would need from a training package in order to confidently deliver the service.

Stage 4: A review event was held to thank workshop attendees who had contributed and celebrate what we had achieved. Achievements included the successful facilitation of constructive dialogue among diverse stakeholders, the timely development of an ACF service prototype through co-design processes, and the generation of valuable insights that informed our trial model. Despite the evening scheduling, the workshops maintained strong attendance and produced the necessary outputs to advance the design process. The facilitated discussion focused on reviewing the co-designed ACF service and identifying outstanding uncertainties. Attendees were also asked to reflect on their experience of co-design and share any aspects they had learned or would take from the experience. This reflection allowed participants to consolidate their insights and consider how they might apply co-design principles in future projects. This event was attended by fifteen participants (two GPs, two pharmacists, one person with lived experience of CRC and ten community members).

We incorporated participant feedback through multiple channels throughout the study. After each workshop, participants completed feedback forms, and we actively invited verbal input during sessions as part of our co-design methodology. This feedback directly informed the structure of the workshops and iterations of our materials, including information sheets, graphics, posters and translation choices. Between workshops, we maintained engagement through newsletters and email correspondence to facilitate ongoing dialogue. Our commitment to incorporating participant perspectives was fundamental to our design ethos, with challenge and critique actively encouraged. An additional workshop to update on the trial of the ACF service was organised specifically at the request of participants, demonstrating our responsiveness to their needs and interests.

Our approach to developing the ACF service aligns closely with the actions for designing and creating interventions as described by O’Cathain et al. [49]. We engaged in generating ideas through co-design workshops and bringing together diverse stakeholders to creatively develop service components. Key decisions about the content, format, and delivery of the intervention were made collaboratively, resulting in multilingual materials, staff training packages, and communication protocols. We designed an implementation plan that considered integration with existing systems and inter-professional collaboration. Throughout the process, we created and refined prototypes of patient-facing materials based on iterative feedback from workshop participants. This iterative, stakeholder-driven approach ensured that our intervention development was grounded in the needs and perspectives of those who would use and deliver the service.

### Focused ethnography

#### Qualitative approach and research paradigm

To document the ongoing co-design process, as well as to provide timely insights to inform it, we conducted a focused ethnography of the EBCD process [50], consisting of semi-structured interviews (see above, EBCD, Stage 1), observation of online and in-person workshops (EBCD Stages 2–4) and Rapid Qualitative Inquiry (RQI) to collect and analyse qualitative data efficiently without compromising rigour [28]. Traditional ethnography typically involves long-term, immersive fieldwork aimed at understanding entire cultures or communities, often with open-ended research questions and a broad scope. In contrast, focused ethnography involves short-term field visits, intensive data collection, and a focus on a discrete community, making it well suited for studying specific aspects of society and informing interventions [51]. Rapid Qualitative Inquiry follows four steps, two of which iterate: [1] collect initial data through interviews and observations, logging data in shared field notes; (2) analyse data by coding, memo-ing, and discussing emerging patterns and themes as a team; identify gaps and new lines of inquiry; (3) based on the analysis, collect additional data interviews/observations to fill gaps and test emerging conclusions; and,after multiple iterative cycles of Steps 2–3, (4) share tentative results with participants to get feedback and validation, incorporating their input into conclusions. Our research philosophy is pragmatic in nature [52], being: problem-orientated (seeking practical solutions), anti-foundational (seeing knowledge as evolving and contingent); community-based (embedded in a group of inquiring patients and clinicians); context-aware (concerned with the tension between different felt needs); consequentialist (mindful of the practical utility of prioritising certain understandings over others; and, fallibilist (our findings are open to revision).

### Researcher position

During the workshops, three researchers—each experienced in qualitative research with underserved communities—were positioned as participant-observers. They were introduced to participants as researchers interested in understanding the co-design process. The researchers built trust with participants through prolonged engagement over five months during and in-between workshops by adopting an invitational communication style that encouraged constructive check and challenge. This approach was essential for stress-testing ideas, facts, and perspectives while welcoming additional insights, facilitated through personal relationships maintained in person, over the phone, and by email. This trust-building was particularly important with underserved communities who expressed anxiety and hesitation when interacting with institutional entities such as the NHS.and by maintaining a non-judgmental, facilitative stance. The research team included a regional research ambassador, who is experienced in engaging with underserved and marginalised communities. Inclusion of this individual on the research team also helped build trust and legitimacy with these communities. One researcher was seated at each table alongside community members, healthcare professionals, and other stakeholders. This allowed the researchers to take field notes capturing detailed observations on interactions and discussions while actively participating in the workshop, building rapport with other participants, without disrupting the collaborative environment.

### Data collection

Field notes were used to document views of attendees, notable interactions and aid service development. Alongside capturing real-time observations during workshop activities, researchers and participants naturally engaged in informal conversations during breaks and between workshops. While these interactions helped build rapport and trust, they were not treated as formal data collection opportunities. All structured research activities were conducted during dedicated workshop sessions with participants’ full awareness and consent. Field notes were recorded during and immediately after each workshop. In line with best practice [28], we triangulated interview content and field notes with other data sources, Additional data included artefacts from the workshops (e.g., post it notes, training materials, and patient facing materials generated by workshop participants).

### Analysis

**Fig. 1:**
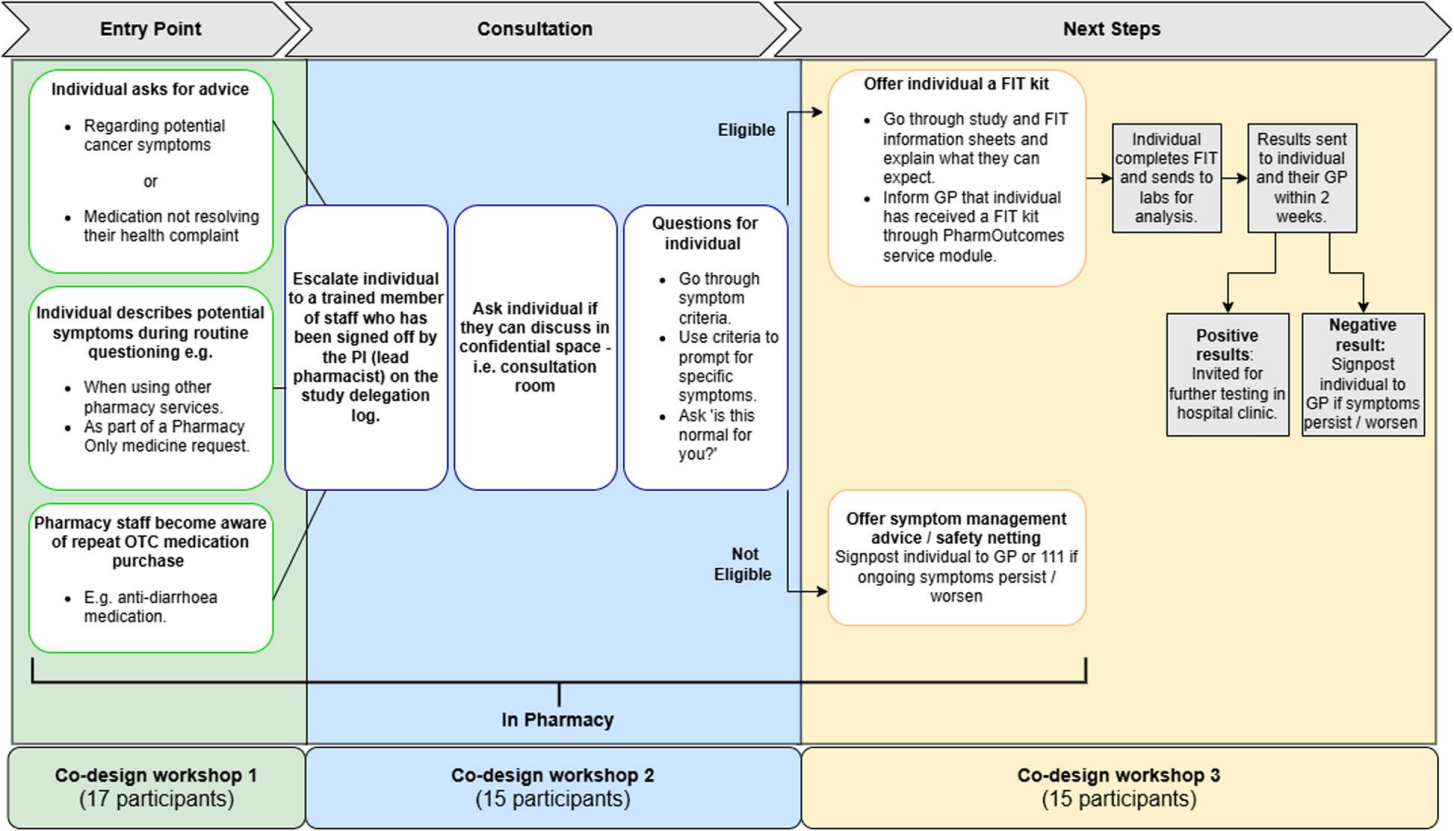
Schematic representation of ACF service pathway. Pathway is based on NHS England’s model for pharmacy-based detection [29]. Co-design workshops followed the chronological patient journey, enabling comprehensive stakeholder input at each critical transition point in the service pathway

After each workshop, researchers independently analysed the notes, identifying potential themes and patterns. Analysis began with immersion in field notes consisting of iterative reading and annotation. This then expanded to the wider dataset including workshop artefacts (‘post-it’ notes, worksheets), summarising observations and organising them to develop themes [50] (Additional Files 5 and 6). Following initial individual analysis, we convened in team meetings to discuss and synthesise observations and refine identified key themes along the section of the pathway discussed at that session (see Fig. 1). Researchers identified key themes that would shape the service co-design. Attention was paid to points of tension in the proposed service pathway. The research team’s insider knowledge from workshop involvement enabled them to provide context, but also required reflexivity to foreground participant perspectives.

We developed a standardised summary template organised around key domains of interest, enabling rapid synthesis of workshop data. Analysis followed an iterative cycle, with insights from each workshop guiding inquiry in subsequent sessions. We carried out data condensation, which involved coding field notes to identify thought units and applying initial codes. The research team engaged in margin note-taking to record reactions, interpretations, and insights during the coding process. Thematic matrices were used to organise the coded data and support drawing conclusions [28].

In alignment with Beebe’s description of RQI as an open system that encourages rapid changes to questions, topics, and direction as new information is gathered (p. 82 [28]), field notes were reviewed and discussed extensively by the research team between workshops to identify emerging themes and areas requiring further exploration.

#### The production of programme theory and a logic model

The focused ethnographic approach employed in this study enabled us to uncover key cultural/systems-level considerations for community pharmacy-based equity-focused interventions in underserved areas. The rapid qualitative analysis aimed to produce overarching principles, generalisable beyond the specifics of our co-design process, aligning with focused ethnography’s aim to examine particular aspects of society to address practical problems [50]. The generation of these insights also had a concrete goal of designing a context-specific active case-finding service; it was crucial to ground practical application in the rich understanding provided by the focused ethnographic analysis [51]. The MRC Framework states that logic models and programme theories are essential for clarifying how complex interventions produce change and should guide all stages of service design and evaluation [53]. Programme theory explains how and why an intervention is expected to work by articulating the relationships between strategies and intended results, grounded in research, practice, and theory [54, 55]. Logic models visually represent this theory to support design, planning, communication, learning, evaluation, stakeholder engagement, and context-sensitive implementation [54–57]. Therefore, as a companion to our co-designed service model, we developed a candidate programme theory of the ACF service as one of the concrete “deliverables” which a focused ethnography should have [50], demonstrating how its collaboratively generated and locally grounded can be operationalised into practice. The programme theory of the ACF service, aimed to express the needs of the community it aims to serve, using an “if… then… so that… so that…” framework to articulate how specific service components are expected to produce desired outcomes in underserved communities [53]. We used this theory to develop the ACF service. Alongside the schematic representation of the ACF service pathway (Fig. 1), we expressed the programme theory as a logic model mapping the relationships between resources, activities, outputs, and outcomes, providing a visual representation of how the service is expected to function and achieve its goals. We also described the essential components of the ACF service using the Template for Intervention Description and Replication for Population Health and Policy interventions (TIDieR-PHP) framework, a structured reporting guideline designed to improve the completeness and replicability of public health intervention descriptions in research [58].

#### Techniques to enhance trustworthiness

The focused ethnography took place over five months with four co-design workshops, allowing sufficient time for several cycles of data collection and analysis, in line with best practice for rapid qualitative inquiry [28]. We triangulated data from multiple primary sources (interviews, workshop observations, notes and artifacts), held regular team meetings to discuss interpretations. During the workshops and the team meetings and analysis sessions between them, RM played the role of a peer debriefer, regularly reminding us of our positionality, encouraging us to engage with alternative perspectives through discussing and checking interpretations and acknowledging power dynamics [59]. We conducted member-checking [60–62] with workshop participants at subsequent sessions, to assess our interpretation of their contributions, asking: “have I understood correctly what you wanted me to know?” Our proposed themes were shared with participants to verify their accuracy and authentic representation of the workshop discussions. Participants were invited to provide feedback, suggest modifications, or highlight any potential misinterpretations, enhancing the reliability of our interpretations. Finally, we grounded our findings and interpretations through recourse to the peer review literature, to ensure we had captured different multiple ways.

of understanding the topic [59]; in our results, we annotate components of our programme theory with citations from other contexts that allow the reader to see these points of triangulation.

#### Ethical concerns

The University of Sheffield, Department of Psychology Research Ethics Committee, granted ethics approval (reference: 057720). Workshop attendees were informed that researchers would collect field notes throughout the event. Those who did not want their contributions recorded could opt out by completing a form or notifying researchers directly. No participants chose to opt out at any point. We implemented several safeguards to protect participants’ inputs, including this opt-out consent process that allowed individuals to decline observation without exclusion from workshops. All field notes were pseudonymised. Workshop products, including service designs and training materials, were reviewed by participants with sufficient time for amendments prior to implementation. Given the sensitive nature of colorectal cancer discussions and the diversity of our participant group, we prioritised creating a safe environment where contributions could be made without fear of identification in subsequent publications.

## Results

Three overarching themes were developed based on discussions during the course of the co-design workshops: 1) amplifying community pharmacy assets, 2) strengthening inclusive practice, and 3) enabling service integration and quality. These themes were identified through our collaborative thematic interpretation approach, where field notes and workshop artifacts underwent iterative analysis and coding by multiple researchers. The thematic matrices created during our data condensation process enabled us to recognise these key patterns across the dataset. Each of these themes encompassed several sub-themes which highlighted key considerations in the service design and shaped development of the ACF service. To ensure the themes accurately reflected attendees’ experiences of the co-design process, researchers conducted member checking with participants by presenting the identified themes and sub-themes during the final workshop and inviting feedback on our interpretation.

### Amplifying community pharmacy assets

#### Pharmacy accessibility can increase public engagement about bowel cancer

*Community pharmacies already often serve as accessible healthcare venues and as first points of contact for health concerns (Resources)* [16]*. This accessibility could allow pharmacists and counter staff to actively engage with community members about bowel health (Activities)* [14, 63]*.*

Community pharmacies were understood to be highly accessible healthcare venues. Participants frequently described pharmacies as community hubs, often serving as the first point of contact for health-related concerns. A community member shared, *“At the end, I had a good pharmacist – and he was really concerned about how I looked” (patient during workshops).* This sentiment was echoed by healthcare professionals, with one GP acknowledging the increasing utilisation of pharmacy services.

However, the co-design process also revealed a gap in public awareness about the scope of pharmacy services. Some community members were unaware that pharmacists could provide health advice or that certain symptoms warranted discussion. As one community member noted, *“Why would I open this conversation with a pharmacist—I will just call my GP” (community member during workshops).* A pharmacist also highlighted that *“There is a misconception that we just sell stuff over the counter… we are trained, regulated and go through continued development” (pharmacist during workshops).* In response, participants co-designed promotional posters advertising the ACF service, with visual representations of target symptoms, for display in participating pharmacies. Additionally, we implemented multi-faceted outreach strategies including direct engagement with GP practices across the region to increase awareness of the pharmacy service. We also conducted outreach sessions with diverse community groups throughout South Yorkshire, leveraging existing community hubs and networks to disseminate information about the service.

#### Pharmacies must build trust and relationships

*Clear information and support, throughout the testing process, (Activities) are required for trust in pharmacy-based health services to increase (Outputs)* [64].

The workshops emphasised the critical role of trust in healthcare interactions, particularly for marginalised groups [65]. Pharmacies were identified as well-positioned to build and maintain trust within communities. Pharmacists noted that they often see the same individuals regularly, giving them the opportunity to build relationships. This humanistic approach would be key in the ACF service, which may rely on these relationships to recognise eligible individuals and determine the most effective way to engage with them.

Supporting this trust-building capacity is the robust professional infrastructure within pharmacy practice. Workshop discussions revealed that pharmacies operate under qualified pharmacists who adhere to strict professional standards, with mandatory continuing professional development requirements. This clear accountability structure, combined with established protocols for patient confidentiality and direct communication with primary care provides a foundation for community trust in pharmacists’ authority to deliver services.

A pharmacist also raised the importance of follow-up, asking, *“Will someone follow them up? They’ve got to trust that something will be done” (pharmacist during workshops).* This was reinforced by a community member who remarked, *“It’s for us to put our trust in pharmacists” (community member during workshops).* This highlights the importance of establishing and communicating well-defined follow-up protocols for the ACF service. This was addressed by ensuring both participants and GPs were notified of test results (via letter) and follow up actions, which would be managed in secondary care. Pharmacists were able to clarify this follow up procedure to participants during the consultation process.

### Strengthening Inclusive Practice

#### Private, culturally sensitive consultations are necessary for sensitive symptom discussions

*Private consultation spaces in participating pharmacies” (Resources) are a precondition for individuals with potential symptoms to be offered FIT tests in a private, culturally sensitive manner (Activities), and reducing barriers to discussing sensitive health topics (Outputs)* [66, 67]*.*

Given the potentially embarrassing nature of bowel symptoms, privacy emerged as a crucial concern. Workshop participants stressed the need for private consultation spaces in pharmacies delivering the ACF service. *“It’s about feeling safe, having somewhere private where they can go and be given the FIT…it can be embarrassing,”* explained one community member during the workshops. This was further reinforced by others who expressed discomfort at the prospect of discussing sensitive matters within earshot of fellow community members, highlighting how social dynamics within close-knit communities influence willingness to engage with services. These concerns underscored the importance of providing confidential spaces for consultations. These privacy concerns may extend beyond the physical space limitations to reflect broader systemic pressures within pharmacy services. Workshop discussions revealed how stretched pharmacy capacity can compromise privacy and patient experience. Staffing constraints means privacy may be compromised when patients must wait for pharmacist availability, potentially increasing discomfort around sensitive discussions.

#### Cultural competency is necessary for inclusive cancer detection

*Training pharmacists and counter staff trained in cultural competency and symptom recognition (Resources) is a precondition for offering FIT tests in a culturally sensitive manner (Activities) and reducing barriers to discussing sensitive health topics will be reduced” (Outputs)* [68–70].

Cultural and language considerations were prominent themes throughout the co-design process. Participants discussed varying comfort levels with discussing sensitive health topics across cultures and generations, as well as preferences for healthcare providers of the same sex. Attendees highlighted the importance of using clear non-technical language when engaging with individuals and incorporating visual depictions of at-risk symptoms to aid understanding. In response to these insights, patient-facing materials were co-designed with participants. These materials underwent three iterations, incorporating feedback from participants before finalisation. Feedback led to changes in structure, readability and content of the materials which included graphics produced by participants. To address language barriers, pharmacies interested in participating in the ACF service trial consulted about the languages used in their communities. This information, combined with data from the Office of National Statistics (ONS), led to the selection of Urdu, Arabic, Punjabi, and Polish for translations of the patient-facing materials. Furthermore, to enhance accessibility, easy-read versions of the materials were produced. Additionally, the training specifically addressed understanding cultural sensitivities around discussing bowel health, including guidance on offering same-sex consultations when possible, giving individuals the opportunity to take information sheets home before accepting a test, providing emotional support to address cultural fears around diagnosis, and respecting personal or cultural beliefs that might influence attitudes toward testing. Healthcare providers were also trained in adapting their communication styles to diverse patient populations, including using knowledge of regular customers to determine optimal engagement approaches, offering resources in alternative formats, and utilising visual instructions to improve understanding across language barriers.

These efforts aimed to ensure that the ACF service would be easily understood by a diverse range of community members.

#### Accessible health information is necessary for screening participation

*The development of clear, culturally appropriate information about colorectal cancer symptoms and FIT testing (Resources) are one precondition for clear information and support to be provided throughout the testing process (Activities) in order for more individuals from deprived areas to be screened for colorectal cancer (Outputs)* [71, 72].

Participants emphasised the need for clear, culturally appropriate information about the FIT process. They noted potential misconceptions about cancer diagnosis and treatment that needed to be addressed. The workshops revealed that some individuals might not understand the difference between screening and diagnostic tests, or that a positive FIT result would require further investigation and did not equate to a cancer diagnosis. These insights led to the development of a comprehensive FIT information sheet in collaboration with workshop attendees.

In response to these findings, the training specifically equipped providers with communication strategies to effectively explain the FIT process, emphasising techniques to distinguish between screening and diagnostic testing in accessible language. Healthcare providers were trained to proactively address common misconceptions, manage patient anxiety about potential results, and navigate the stigma often associated with bowel conditions. The training included example scenarios for identifying potentially eligible people for the service and clarifying that a positive result indicates the need for further investigation rather than confirming a cancer diagnosis—a distinction workshop participants had identified as particularly confusing. By preparing healthcare providers with these specific communication skills, the service aimed to complement the written information materials and provide consistent, sensitive support throughout the FIT process.

#### Addressing emotional barriers is necessary to improve screening uptake

*Clear information about colorectal cancer symptoms and FIT testing, that considers emotional and psychological factors (Resources) is an essential precondition of a supportive testing process (Activities), such that trust in pharmacy-based health services will increase (Outputs)* [11].

Discussions revealed anxiety and fear associated with the term ‘cancer’, leading to debates about whether to use the word in any study materials. Participants also expressed concerns over fragmented result reporting and lengthy waiting times, highlighting the need for clear communication about the process, timelines, and next steps to alleviate anxiety.

The workshops captured diverse emotional responses across different patient groups. For some participants, discussions about cancer were considered especially sensitive, with one workshop attendee noting that older generations often *“keep a lot to themselves”* regarding health concerns. Cultural perspectives varied significantly: some community members expressed that they would avoid engagement entirely if cancer was mentioned, while others felt the term created necessary urgency and motivation to complete the test.

Patient feedback revealed varied psychological barriers beyond terminology fears. Several participants expressed what one called *“the disgust factor”* associated with handling stool samples, which presented a substantial deterrent to completing the FIT.

These insights from diverse patient perspectives guided the development of a conversation guide that acknowledged varying comfort levels with cancer discussions while providing clear, reassuring information about the testing process and results (Additional File 7).

### Enabling service integration and quality

#### Collaboration with GPs is necessary to for pharmacist ACF

*A system for collaboration between pharmacies and GPs must be established (Resources)* [73] *for test results to be promptly communicated to both patients and their GPs (Activities) such that coordination between pharmacies and GPs in cancer detection is seen to improve (Outputs).*

A key theme that was developed based on our discussions within the workshops was the need for a unified approach between pharmacies and GPs, and clarifying the role of a pharmacy ACF service in relation to existing GP services. Participants emphasised the importance of portraying inter-professional collaboration rather than rivalry, with pharmacies and GPs working together rather than in silos. Concerns were raised about safety netting, especially for negative FIT results. As one GP noted, “*Once the pharmacist has given a FIT out, you wouldn’t want to lose that patient*” *(GP during workshops).* GPs stressed the need to be informed if their patient had been given a FIT and of their subsequent test results. To address these concerns, we collaborated with GPs to co-design letters informing them of their patients’ results. This approach ensured that GPs were kept in the loop and could provide appropriate follow-up care. Additionally, we integrated the ACF service into existing pharmacy software systems, streamlining the process and enabling automatic notifications to GPs when their patients are given a FIT kit.

Our final review event identified outstanding uncertainties, primarily concerning the lack of a follow-up mechanism for unreturned FIT kits. GP participants noted that their standard practice includes sending text reminders to patients who receive FITs through primary care, and they recommended implementing a comparable system for the ACF service. In response, we have established an automated text reminder that is dispatched to all participants one week after they receive a FIT through the ACF service to promote test returns. The content of this reminder message was collaboratively developed with workshop attendees through email correspondence.

#### Comprehensive staff training is necessary for effective community engagement

*Pharmacists and counter staff must receive training, and materials, in cultural competency and symptom recognition (Resources) to actively engage with community members about bowel health (Activities), such that more individuals from deprived areas will be screened for potential colorectal cancer (Outputs)* [64, 74]*.*

The co-design process identified specific qualities, knowledge, and skills required for pharmacists to deliver the ACF service effectively. Through detailed workshop discussions, we identified nine critical training domains essential for successful service implementation:

1. Bowel cancer knowledge, including understanding risk factors and symptoms.
2. Detailed understanding of FIT test specifics, including purpose, sample collection, and result interpretation.
3. Patient communication strategies, particularly for discussing sensitive health topics.
4. Clinical skills for assessing patient eligibility and recognising high-risk symptoms.
5. Ethical and legal considerations, including patient confidentiality and consent.
6. Referral pathways and understanding local healthcare resources.
7. Quality assurance and documentation protocols.
8. Familiarity with any digital systems used for recording consultations.
9. Cultural competence in discussing bowel health with diverse populations

Interestingly, participants suggested that counter staff, not just pharmacists, could play a role in identifying at-risk individuals due to their consistent presence and potential cultural knowledge. This led to discussions about how to operationalize various elements of the program, including modifying existing health IT infrastructure and utilising systems already in place in pharmacies. We worked with pharmacists to develop a training package which encompassed the specific knowledge and skills required. This included slides, case-studies, e-modules and a self-assessment questionnaire to test understanding. The ACF service was also incorporated onto existing pharmacy software (PharmOutcomes) to streamline introduction of the service. The training approach acknowledged the extensive existing expertise of pharmacy staff, focusing instead on identifying and addressing the specific areas of service delivery that the staff themselves had prioritised as most critical to their professional practice.

### ACF service development

The co-design process ensured that the resulting ACF service was grounded in the needs and perspectives of the community it aims to serve. During the review event participants reflected that they “felt valued and very much part of the whole process”, with one pharmacist stating that they “wished all community pharmacy services started like this”.

**Fig. 2:**
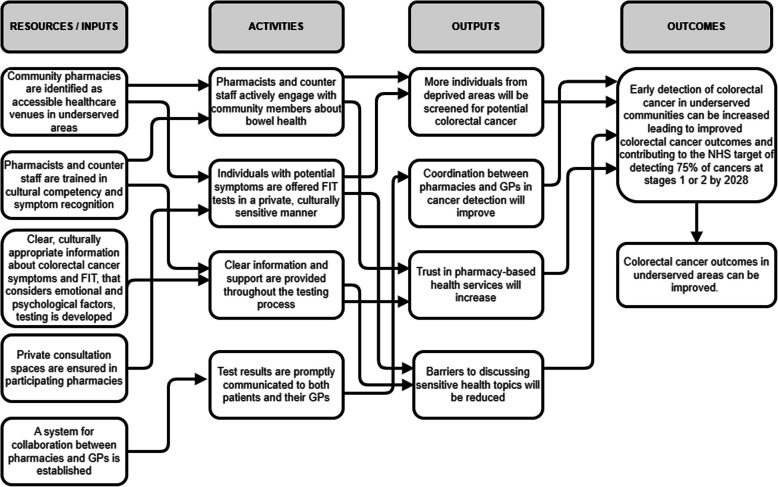
Logic model of ACF service

Table 1 presents the candidate programme theory of the ACF service using an ‘if–then-so that’ framework to articulate how specific service components are expected to produce desired outcomes in underserved communities. We used this theory to develop the ACF service. Figure 1 shows a schematic representation of the ACF service pathway whilst also indicating how each of the three co-design workshops aligned with a particular part of the service development (Fig. 1). The co-design process ensured that the resulting ACF service was grounded in the needs and perspectives of the community it aims to serve. Figure 2 illustrates the programme theory as a logic model mapping the relationships between resources, activities, outputs, and outcomes, providing a visual representation of how the service is expected to function and achieve its goals. Table 2 describes the ACF service using the TIDieR-PHP framework (Template for Intervention Description and Replication for Population Health and Policy interventions), a structured reporting guideline designed to improve the completeness and replicability of public health intervention descriptions in research [58].

**Table 1:** Candidate programme theory of ACF service

**If**
Community pharmacies are identified as accessible healthcare venues in underserved areas
Pharmacists and counter staff are trained in cultural competency and symptom recognition
Clear, culturally appropriate information about colorectal cancer symptoms and FIT testing, that considers emotional and psychological factors, is developed
Private consultation spaces are ensured in participating pharmacies
A system for collaboration between pharmacies and GPs is established
Pharmacists and counter staff actively engage with community members about bowel health
Individuals with potential symptoms are offered FIT tests in a private, culturally sensitive manner
Clear information and support are provided throughout the testing process
Test results are promptly communicated to both patients and their GPs
**Then**
More individuals from underserved areas will be tested for potential colorectal cancer
Barriers to discussing sensitive health topics will be reduced
Trust in pharmacy-based health services will increase
Coordination between pharmacies and GPs in cancer detection will improve
**So that**
Early detection of colorectal cancer in underserved communities can be increased
Colorectal cancer outcomes in underserved areas can be improved, contributing to the NHS target of detecting 75% of cancers at stages 1 or 2 by 2028

**Table 2:** ACF service description within the TIDieR-PHP framework [58]

Brief name	Pharmacy-based Active Case-Finding (ACF) Service for Colorectal Cancer
Why	The ACF service aims to increase early detection of colorectal cancer in underserved communities by leveraging the accessibility and trust of community pharmacies. This addresses health inequalities in cancer outcomes and supports the NHS target of detecting 75% of cancers at stages 1 or 2 by 2028
What materials	- Informational materials about colorectal cancer symptoms and FIT testing (including translations and easy-read versions) - Pictorial representations of at-risk symptoms - FIT kits - Private consultation room in pharmacies - Training materials for pharmacists and counter staff - IT infrastructure for recording and sharing patient data
What and how	- Pharmacists and counter staff actively engage community members about bowel health - Individuals with potential symptoms are offered FIT tests in private consultations - Clear information and support are provided throughout the testing process - Test results are promptly communicated to both patients and their GPs - Referral pathways are established for positive test results
Who provided	- Community pharmacists and counter staff (trained in cultural competency and symptom recognition) - General Practitioners (for follow-up and referrals) - Local health authorities (for oversight and coordination)
Where	Community pharmacies in underserved areas of Yorkshire, UK. The socioeconomic context of these areas, including lower life expectancy and higher deprivation levels, is relevant to the intervention’s design and implementation
When and how often	The service is intended to be ongoing, with pharmacies offering the ACF service during regular opening hours. Individual engagement frequency will vary based on community needs and pharmacy capacity
Planned variation	The service allows for tailoring to local needs, including: - Provision of materials in languages common to specific areas - Flexibility in how pharmacies integrate the service into their existing workflows - Adaptation of engagement strategies to suit local cultural norms and preferences
How well (strategies to maintain fidelity)	- Comprehensive training program for pharmacists and counter staff - Regular monitoring and feedback on service delivery - Establishment of clear protocols and guidelines for implementing the ACF service - Ongoing support and resources for participating pharmacies

## Discussion

We developed a pharmacy-based ACF service for colorectal cancer detection in underserved areas of Yorkshire, UK, using an EBCD approach. The resulting service model leverages the accessibility and trust of community pharmacies to increase early detection of colorectal cancer in underserved communities. This modified EBCD approach was strategically chosen to mitigate the potential pitfalls often associated with top-down imposed initiatives. By engaging stakeholders early in the process, we aimed to limit the need for extensive rework that can arise when services fall short of meeting the needs of their intended beneficiaries. Additionally, this collaborative approach allowed us to anticipate and address potential delivery challenges earlier in the development process, enhancing the likelihood of creating a service that is both effective and feasible in real-world settings.

### Strengths and limitations

Our study’s key strengths are its originality in applying interdisciplinary co-design methodology to develop a novel pharmacy-based CRC ACF service; it is significant in addressing health inequalities and promoting early detection in underserved communities. Additionally, it is rigorous in employing a robust mixed-methods design that spans health services research, primary care, and community engagement.

The use of a modified EBCD approach engaged a diverse range of stakeholders including pharmacists, GPs, patients, and community members. The workshops were well attended, with an average of 15 participants at each session. Notably, 12 individuals attended at least three workshops, demonstrating a high level of repeat engagement and commitment to the co-design process. The presence of an independent facilitator provided a neutral voice, which fostered constructive dialogue among diverse stakeholders. This participatory method ensured that the service design was grounded in the needs and perspectives of those who will use and deliver it. As a result, participants felt a sense of ownership for the developed service and there was a strong desire to remain involved with how the service progresses during the trial. The concurrent focused ethnography provided rich insights into the collaborative sense-making process underlying the final service design.

However, the study has limitations. The co-design process was conducted in a specific region of the UK, which may limit the generalisability of the findings to other contexts. Additionally, while we engaged a range of stakeholders, the views captured may not be representative of all potential service users or providers. Resource constraints meant co-design work was limited to 4 meetings, which restricted the depth and breadth of stakeholder engagement and collaboration within the co-design process. Researchers had to rely on email communication between workshops to maintain engagement.

A specific limitation resulting from these resource constraints was our inability to develop culturally adapted interventions. While we identified different adaptation needs for diverse communities during the workshops, we lacked the time to implement these adaptations. Consequently, we adopted a broader cultural competency approach rather than producing tailored materials for specific communities. With additional meetings and resources to work directly with these communities, we could have created more focused, culturally appropriate materials.

Future co-design research may consider ensuring sufficient resources for more frequent and regular meetings with stakeholders to enhance the depth and continuity of collaborative efforts.

Given that this service was co-designed for a feasibility trial, we also faced constraints in what could be achieved within the limited time frame. One notable example was the concern raised by GPs regarding the absence of a process to follow up on unreturned FIT kits (enabling service integration and quality). We did not incorporate this feature into the ACF service for two reasons: firstly, to avoid overburdening pharmacies, which had previously expressed capacity limitations, and secondly, to prevent adding to GPs’ workload through the introduction of the service. Consequently, the rate of unreturned FIT kits will serve as a key outcome measure in the subsequent trial of the service.

### Wider context

Our findings advance previous research by addressing critical gaps in pharmacy-based cancer detection. Our study moves beyond the feasibility of ACF, documented by systematic reviews [75, 76], feasibility to examine effectiveness in underserved communities specifically. Our co-designed ACF service anticipates and responds to recent calls for pharmacy interventions with an explicit health inequalities lens, employing a ‘disadvantaged groups approach’ that focuses on culturally appropriate materials, privacy considerations, and trust-building mechanisms [77]. This represents a significant advancement in pharmaceutical public health approaches that aim to improve early cancer detection while addressing health inequalities.

Our study builds on previous research demonstrating the potential of pharmacy-based interventions for cancer screening. For instance, Lindsey et al.’s systematic review suggested that community pharmacies could significantly contribute to cancer education and screening [76]. Our study extends this work by developing a specific, co-designed service model for colorectal cancer detection in underserved areas.

Unlike studies that focus solely on feasibility, we have emphasised the importance of cultural competency and addressing barriers specific to underserved communities such as language, health literacy and mistrust, which exacerbate the association between socio-economic deprivation and poorer CRC outcomes [7, 78]. The recent NHS Race and Health Observatory report, has reinforced the barriers health services face in gaining the trust of patients with alarm symptoms, which directly impacts screening participation and early cancer detection [11].

### Implications for clinicians and policymakers

This study demonstrates the potential of community pharmacies to play a pivotal role in addressing health inequalities, particularly in cancer detection. The ACF service model could provide a mechanism for increasing early detection of colorectal cancer in underserved areas, contributing to the NHS Long Term Plan’s ambition of diagnosing 75% of cancers at stage 1 or 2 by 2028 [24]. Furthermore, our co-designed model presented here offers a template that could extend beyond CRC detection to other conditions, where early detection is crucial and health inequalities persist.

For clinicians, particularly GPs and pharmacists, this study highlights the importance of inter-professional collaboration in cancer detection. It also emphasises the need for cultural competency training to effectively engage with diverse communities. Our findings also suggest that pharmacy counter staff can play a valuable role in proactively identifying potential service users, which has implications for pharmacy workflow and staff responsibility allocation.

For policymakers, our findings suggest that investing in pharmacy-based health interventions could be an effective strategy for addressing health inequalities. The study also underscores the value of co-design approaches in developing healthcare services that are acceptable and feasible for both providers and users [79]. Robust health information systems should also be prioritised, which would allow seamless integration between pharmacy and primary care records and facilitate collaborative care pathways.

### Unanswered questions and future research

The next step in this research programme is a cluster randomised controlled trial comparing ACF for colorectal cancer in community pharmacies to usual care (ISRCTN: 14,156,362). This pilot trial will randomise 40 Lower Layer Super Output Areas (LSOAs) in South Yorkshire’s most deprived areas. The primary feasibility outcome is distribution of 1000 FITs from 20 pharmacies over 12 months. Secondary outcomes include intervention adherence and stakeholder views on acceptability. It will also provide preliminary data on FIT uptake, cancer detection rates, and stage at diagnosis.

Based on health economic modelling, we can estimate the potential impact of our ACF intervention on colorectal cancer outcomes. Assuming 1000 FIT kits are distributed from 20 pharmacies (tested at the same threshold currently being used in GP primary care symptomatic services), we anticipate detecting approximately 10 colorectal cancers [80]. Of these, 4 would likely be early-stage (Stage I or II) diagnoses that might otherwise have been detected at a later stage without the intervention. The modelling suggests that expediting diagnosis by several months could lead to significant survival benefits. For the 3 patients with Stage IV cancer, an earlier diagnosis might extend survival by an average of 9 months [81]. More importantly, for the 7 patients with Stage I-III cancers, the earlier diagnosis could translate to long-term survival benefits extending beyond 5 years [81]. Additionally, the intervention is expected to identify 30 individuals with high-risk polyps (defined in accordance with BSG guidelines) [82]. Based on previous research, removing these polyps could reduce mortality by 53% in this group [83]. Combining these factors—earlier diagnosis of cancers and removal of high-risk polyps—the model suggests that this intervention could potentially save 12 lives over the long term.

If the intervention and study appear feasible and scalable, future research will explore the transferability of this service model to other geographical areas. Long-term studies will be needed to determine whether this approach leads to improved colorectal cancer outcomes in underserved communities.

## Conclusion

In conclusion, this study provides a co-designed model for pharmacy-based ACF of colorectal cancer in underserved areas. While further research is needed to evaluate its effectiveness, the model shows promise in addressing health inequalities and improving early cancer detection in underserved communities. Furthermore, this study has demonstrated how co-design methodology can improve professional understanding of underserved communities, which can strengthen future practice in addressing health disparities.

## Supplementary Information

Additional file 1. GRIPP2 reporting checklist

Additional file 2. Semi-structured interview topic guide

Additional file 3. Breakdown of participants attending each workshop

Additional file 4. Table to show questions/prompts used in each of the workshops

Additional file 5. Table handout for materials review

Additional file 6. Workshop themes

Additional file 7. Conversation guide

## Data Availability

Workshops and informal interviews were not recorded; however, notes taken during the workshops and interviews are available from the corresponding author on reasonable request.

## Abbreviations

CRC: Colorectal Cancer
ACF: Active Case Finding
FIT: Faecal Immunochemical Test
EBCD: Experience Based Co-Design
ONS: Office of National Statistics
LSOAs: Lower Layer Super Output Areas
