# Supplementary material for: Experience-based co-design of an active case finding service for colorectal cancer in community pharmacies: findings from a focused ethnography
Source: Res Involv Engagem. 2025 Jun 10;11:59. doi: 10.1186/s40900-025-00740-0 (PMC12150438; doi:10.1186/s40900-025-00740-0)
Supplement: Supplementary file 2 — Additional file 2. Semi-structured interview topic guide [file 40900_2025_740_MOESM2_ESM.pdf]

Interview Topic Guide

Welcome and Purpose:
- Introduce yourself/others and the purpose of the informal interviews
- Briefly explain the format and duration of the interview
- Emphasise no naming of any individuals, but instead to identify content/ themes as stimulus for future workshops in 2024.

Experience and Expertise:
- Explore the participant's experience or expertise of (bowel) cancer and role of early detection.
- Explore the participant's experiences, opinions, or observations
- Discuss role of improving inclusion and accessibility in under-served populations

Emerging areas that are relevant to DETECT-CRC:
- Discuss the widening adoption of pharmacy interventions and relevance to DETECT-CRC.
- Explore thoughts on these additional pharmacy roles - consider environment/ autonomy in held discussions.
- Explore communication that currently exists between community pharmacies and GPs (eg BP/ glucose checks).
- Discuss C-the-Signs and identify whether use in pharmacies would have any benefits.
- Discuss the current bowel cancer screening model, and explore if parallels could exist in delivering a symptomatic FIT service outside of GPs (DETECT-CRC). What are the potential barriers? What are the potential facilitators?
- What are the key steps to identifying relevant at-risk people in pharmacies? How does it work for other interventions?
- Explore thoughts on governance. Who should have responsibility?

Encourage thoughts that may not have been covered in the predefined themes.
- Unanticipated Insights:
- Probe for unexpected or surprising perspectives that may have emerged during the interview.

Predictions and Expectations:
- Discuss the participant's expectations or predictions regarding future development of DETECT-CRC
- Explore any concerns or aspirations they may have.

Conclusion:
- Allow participants to provide feedback on the informal interview process.
- Invite any final thoughts or comments they may have.

Closing - Thank You and Next Steps:
- Express gratitude for the participant's time and insights.
- Provide information about follow-up steps, if applicable.
