# Supplementary material for: Experience-based co-design of an active case finding service for colorectal cancer in community pharmacies: findings from a focused ethnography
Source: Res Involv Engagem. 2025 Jun 10;11:59. doi: 10.1186/s40900-025-00740-0 (PMC12150438; doi:10.1186/s40900-025-00740-0)
Supplement: Supplementary file 3 — Additional file 3. Breakdown of participants attending each workshop [file 40900_2025_740_MOESM3_ESM.pdf]

Additional file 3: Table to show numbers and types of participants attending each workshop.

Participant	Attendance				
	Introductory online co-design meeting	Co-design workshop 1	Co-design workshop 2	Co-design workshop 3	Review event
**Voice of Intent (enthusiasts and people with authority/mandate to drive change)**					
South Yorkshire and Bassetlaw cancer alliance primary care clinical lead / GP	Yes	Yes		Yes	Yes
South Yorkshire integrated care board community pharmacy clinical lead.	Yes	Yes	Yes		Yes
NHS England cancer programme - senior strategy manager	Yes				
**Voice of Experience (patients/community members*)**					
Lived experience representative	Yes	Yes	Yes	Yes	Yes
Community member	Yes	Yes	Yes	Yes	Yes
Community member	Yes	Yes	Yes		Yes
Community member	Yes	Yes	Yes	Yes	Yes
Community member		Yes			
Community member		Yes	Yes	Yes	Yes
Community member		Yes	Yes	Yes	Yes
Community member		Yes	Yes	Yes	Yes
Community member		Yes	Yes	Yes	Yes
Community member		Yes	Yes		
Community member		Yes	Yes	Yes	Yes
Community member				Yes	
Community member				Yes	Yes
Community member				Yes	
Community member			Yes	Yes	Yes
**Voice of Capability (pharmacists, GPs)**					
Doncaster GP	Yes				
Barnsley GP				Yes	Yes
Barnsley pharmacist	Yes	Yes			
Chief pharmacist of regional pharmacy chain	Yes	Yes	Yes	Yes	
Managing director of Sheffield pharmacy	Yes	Yes			Yes
Sheffield pharmacist	Yes				

Sheffield pharmacist	Yes	Yes	Yes		
Secondary care service manager			Yes		

* Community members were invited through a range of community organisations within target areas of South Yorkshire. These included Healthy Her (Muslim women's health group), Sheffield African Caribbean Mental Health Association (SACHMA), Ethnic Minority Research Inclusion (EMRI), Deep End patient and public involvement network and Sheffield and district African Caribbean community association (SADACCA).
