# Supplementary material for: Experience-based co-design of an active case finding service for colorectal cancer in community pharmacies: findings from a focused ethnography
Source: Res Involv Engagem. 2025 Jun 10;11:59. doi: 10.1186/s40900-025-00740-0 (PMC12150438; doi:10.1186/s40900-025-00740-0)
Supplement: Supplementary file 4 — Additional file 4. Table to show questions/prompts used in each of the workshops [file 40900_2025_740_MOESM4_ESM.pdf]

Additional File 4 – Table to show questions and prompts used in each workshop to guide discussion.

Workshop	Questions / Prompts
Introductory co-design event	**Introductions:**<br>• "Your Name."<br>• "The experience you bring today."<br>• "One thing you'd like to take from this meeting."<br><br>Questions and comments invited about the study and proposed co-design approach.
Co-design workshop 1	**Focused on the types of people who may approach the service and the barriers they may face.**<br>• How accurately do these personas reflect the real experiences, fears, and aspirations of the target demographics? Are there stereotypes that we may inadvertently be reinforcing?<br>• How do various aspects of the personas' identities (e.g., age, ethnicity, economic status, migrant status) intersect to affect their health behaviours and access to services?<br>• What unique challenges does each persona face that might not be immediately apparent without considering the intersection of these factors?<br>• Adopt the User's Perspective: Consider the experience from the viewpoint of personas like Aisha, Brian, and Omar. Imagine their journey through the system and document their experiences.<br>• Spot the Touchpoints: At each stage, pinpoint all the interactions the persona could have with healthcare services. No detail is too small.<br>• Track Emotional Responses: Try to empathize with the persona and anticipate their feelings at each touchpoint, noting any potential distress, confusion, or relief.<br>• Identify Friction Points: Look for moments where the persona might encounter difficulties, delays, or misunderstandings.<br>• Detail is Key: Pay attention to all elements of the interaction, as these can reveal opportunities for enhancing the patient experience.<br>• Consider what adaptations users might feel the need make to cope with deficiencies in the service. How could the service adapt?
Co-design workshop 2	**Focused on the consultation process pharmacists would have with potential service users.**<br>• What should the eligibility criteria be for DETECT-CRC? And how should we adapt the existing NICE DG56 criteria for use in pharmacies?<br>• What are the challenges in using the DG56 criteria?<br>• Please turn the criteria into questions that people can respond easily to.

<table>
<tr><td></td><td>

What would a questionnaire look like?
What would a conversation guide for pharmacists look like?
What would a pictorial representation look like, how to tackle communication barriers?
What are the sources of mistrust in the community?
What can we do to address this mistrust?

</td></tr>
<tr><td>Co-design workshop 3</td><td>
Focused on the communication of FIT results and expectations of service.

Provide feedback on draft materials and appropriateness for local communities (materials included posters, information sheets, GP letters and patient results letters).
Discussion towards identifying specific qualities, knowledge, and skills pharmacists should possess to effectively deliver the patient facing element of DETECT-CRC

"What qualities do you believe are most important for pharmacists to have when offering FIT kits in pharmacy?"
"What specific knowledge do you think pharmacists need to effectively assist patients when offering FIT kits in pharmacy?"
"Can you describe any skills that you think are essential for pharmacists when offering FIT kits in pharmacy?"

</td></tr>
<tr><td>Pharmacy training workshop</td><td>
Focused on exploring the training needs of pharmacy staff.
Checking the following assumptions with pharmacy staff.

Each community pharmacy is run by a qualified pharmacist adhering to professional standards.
Counter staff are supervised by a pharmacist.
Roles, responsibilities, accountability and professional boundaries in each business are explicitly defined and understood by employer and employee.
Training and continuing professional development are mandatory for employer and employees.
Dedicated time is set aside for training and continuing professional development.
Relevant and appropriate competencies are assessed regularly.

Review Proposed Content:

"What's your immediate response?"
"Is there anything missing?"
"Is there anything that is unnecessary?"
"Any other reflections?"

Resources and Capacity:

"Who will deliver the training for DETECT-CRC?"
"How will training be delivered? For example online or in person."
"When does training need to be delivered?"
"What needs to be covered?"

</td></tr>
</table>

<table>
<tr><td></td><td>

"Who will develop content?"
"Will there be a quality assurance process?"
"How will the impact of training be assessed?

PharmOutcomes:

"What data must be collected through PharmOutcomes?"
"What data could be collected through PharmOutcomes?"

</td></tr>
<tr><td>Co-design review event</td><td>
Progress Review and Reflection

Questions about ongoing and new risks for this work and how these can be managed
Reflection questions about the co-design process:

What have you learned?
What have you learned about yourself?
What will you take from this experience?
What will you leave behind from this experience?
One word to sum up how you feel about taking part

</td></tr>
</table>
