# Supplementary material for: Experience-based co-design of an active case finding service for colorectal cancer in community pharmacies: findings from a focused ethnography
Source: Res Involv Engagem. 2025 Jun 10;11:59. doi: 10.1186/s40900-025-00740-0 (PMC12150438; doi:10.1186/s40900-025-00740-0)
Supplement: Supplementary file 5 — Additional file 5. Table handout for materials review [file 40900_2025_740_MOESM5_ESM.pdf]

Tell us what you make of these documents

Working together on the table, take 5 minutes to:

Roles

Briefly introduce yourself by name

Agree:

1 person to make sure every person has an opportunity to contribute

1 person to make sure that the main points are captured with support from others

1 person to feedback up to 3 headlines to the room at 20.10

**Use these questions to guide discussion**	**Use this space to make notes -** (make it clear which document you are referring to).
1. Is there anything that is missing?	
2. Is there anything you think won’t work?	
3. Is there any wording that you think could be made clearer?	
4. What needs changing?	

5. What will make it good enough?	
6.Any other comments?	
