# Supplementary material for: Experience-based co-design of an active case finding service for colorectal cancer in community pharmacies: findings from a focused ethnography
Source: Res Involv Engagem. 2025 Jun 10;11:59. doi: 10.1186/s40900-025-00740-0 (PMC12150438; doi:10.1186/s40900-025-00740-0)
Supplement: Supplementary file 6 — Additional file 6. Workshop themes [file 40900_2025_740_MOESM6_ESM.pdf]

Source	Themes	Quotes	Service output
Workshop 1, 2, 3 and informal interviews.	**Pharmacy accessibility can increase public engagement about bowel cancer**<br><br>Pharmacies are seen as accessible and trusted places in the community.<br>People use pharmacies frequently, not just for health matters - often seen as a community centre.<br>Pharmacies are often the first point of contact for health issues (GP agreement there is increasing utilisation of pharmacy services).<br>Some people are unaware that pharmacists can give advice / unaware that they're symptoms should be of any concern - so wouldn't feel the need to discuss it in a pharmacy.	"why would I open this conversation with a pharmacist - I will just call my GP"." *community member*<br><br>"at the end I had a good pharmacist – and he was really concerned about how I looked" - *patient representative*<br><br>"There is a misconception that we just sell stuff over the counter… we are trained, regulated and go through continued development." - *Pharmacist*	Promotional material co-designed with workshop attendees.<br><br>Role-playing conversations between pharmacists and customers to refine the approach - using open questions, and follow up questions, checking understanding and repeating back answers - conversation guide created.
Workshop 1, 2	**Private, culturally sensitive consultations are necessary for sensitive symptom discussions**<br><br>Need for private consultation spaces in pharmacies.<br>Consideration of potential embarrassment around bowel symptoms.<br>Concerns about being seen or overheard by community members in the pharmacy.	"it's about feeling safe, having somewhere private where they can go and be given the FIT...can be embarrassing." - *community member*	Pharmacies required to have private consultation room
Workshop 1, 3 and informal interviews.	**Collaboration with GPs is necessary to for pharmacist ACF**<br><br>Need for joined-up approach between pharmacies and GPs.<br>Concerns about safety netting, especially for negative FIT results.<br>Clarifying the role of pharmacy ACF in relation to GP services.<br>There is the risk of portraying interprofessional rivalries – trying to design the system working together – not siloed thinking and not a hierarchy - pharmacies and GP working together.	"Once the pharmacist has given a FIT out, you wouldn't want to lose that patient" - GP	GPs will be made aware if service is being trialled in thier area.<br><br>GP made aware if their patient given FIT, and sent copy of thier results.<br><br>Individuals always advised to speak to GP is symptoms persist / worsen.<br><br>Aim is not to increase GP workload.<br><br>GP result letters co-designed with GPs.

Workshop 2, 3	**Cultural competency is necessary for inclusive cancer detection**<br><br>Understanding that some people may prefer to speak to somone of the same sex.<br>Importance of language and communication style (less technical language / translations / easy-read) - ensure translations are non-technical language / culturally appropriate.<br>Varying comfort levels with discussing sensitive health topics across cultures and generations.	[No quotes available]	Mateirals being made available in easy-read and translations of most common langauges in those areas.<br><br>Pictorial representation of at-risk symptoms (co-designed with attendees) to aid understanding.
Workshop 2 and informal interviews.	**Trust and relationship building**<br>Importance of trust in healthcare providers, especially for marginalized groups. Pharmacies in good position to build trust with individuals.<br>Role of community advocates and peer support in promoting the service.<br>Potential distrust of state systems, particularly among immigrants and refugees.<br>Emphasis on a humanistic approach rather than relying solely on leaflets and posters.	"Will someone follow them up? They've got to trust that something will be done." - *Pharmacist*<br><br>"It's for us to put our trust in pharmacists". - *community member*	
Workshop 2, 3	**Accessible health information is necessary for screening participation**<br><br>Need for clear, culturally appropriate information about the FIT test and cancer screening (also visual/pictorial).<br>Importance of addressing misconceptions about cancer diagnosis and treatment.<br><br>Attendees stated how some may feel unsure of what the test required and would want clear information they can take away that would answer their queries including being clear on timelines.<br><br>Clarify the difference between screening and bowel investigations. Some people may not understand that the FIT test is intended to pick up people who might have early signs of bowel cancer and that it won't be a definitive diagnosis. They may not understand that if they show signs, they will need further investigation and even then, they might be diagnosed with other conditions.	[No quotes available]	Patient facing materials - FIT information sheet.

Workshop 2, 4	**Addressing emotional barriers is necessary to improve screening uptake**<br><br>Anxiety and fear associated with the term 'cancer'.<br>Discussion on whether to use the word 'cancer' in materials (potential to cause anxiety vs. create urgency).<br>Importance of providing emotional support throughout the process.<br>Concerns / anxiety about fragmented result reporting and lengthy waiting times.	[No quotes available]	Being clear about the process, timelines, what happens next.
Workshop 4, pharmacy workshop	**Comprehensive staff training is necessary for effective community engagement**<br><br>Identified qualities: compassion, empathy, friendliness, respect for privacy/confidentiality.<br>Knowledge requirements: study details, cultural competency, community knowledge, FIT procedure, GP access information.<br>Skills needed: communication, confidence, navigating language barriers, demonstrating FIT kit use.<br><br>Suggestion that counter staff, not just pharmacists, could identify at-risk individuals.<br>Recognition of counter staff's consistent presence and potential cultural knowledge.<br><br>Recommends ways to operationalise elements of the programme, for instance, through modification of existing health IT infrastructure.	[No quotes available]	Training session content<br><br>Counter staff role with supervision of pharmacist<br><br>Utilising existing systems used in pharmacies - PharmOutcomes.
