# Supplementary material for: Experience-based co-design of an active case finding service for colorectal cancer in community pharmacies: findings from a focused ethnography
Source: Res Involv Engagem. 2025 Jun 10;11:59. doi: 10.1186/s40900-025-00740-0 (PMC12150438; doi:10.1186/s40900-025-00740-0)
Supplement: Supplementary file 7 — Additional file 7. Conversation guide [file 40900_2025_740_MOESM7_ESM.pdf]

<u>**Pharmacy Conversation Guide**</u>

Start the conversation

- Introduce yourself / your role –
- Ask “How is your health?”
- Identify at-risk symptoms e.g. complaints of stomach pain / irregular bowel habits - “Would you like to come into this room to chat about your symptoms?”

In private consultation room

- Explain the DETECT-CRC service and that you would like to ask them some questions about their bowel health - explain that some questions will include asking about going to the toilet.
- Go through the inclusion criteria by asking questions about their symptoms. Probe further when the individual responds with yes/no. E.g. “How often are you going to the toilet at the moment?”, “Have you noticed any unexplained weight loss?” - “Are your clothes fitting any looser than normal?”
- Check the individual’s understanding of each question - e.g. ensure they know what is meant by terms such as constipation.
- Check and repeat back answers to patients.

If eligible for a FIT kit

- Show and explain the FIT kit - how to do it and what it is for.
- Explain that it is not a diagnostic test.
- Give the individual a FIT information sheet and talk through the process of completing the FIT, sending their sample, and receiving their results.
- Explain that their results are only shared with them and their GP.
- Explain that no identifying information will be recorded for the purposes of the research.
- Ask if they would like to take a FIT kit home.
- Ask if they have any questions.

If not eligible for a FIT kit

- Provide general advice and information about managing their symptoms.
- You may wish to signpost individuals to the GP for support with symptoms unrelated to cancer or because you suspect that they may have a health condition other than suspected cancer. These individuals should be managed in accordance with any locally established arrangements and professional obligations.
- People should always be advised to see their GP if their symptoms persist or get worse.
- Advise individual on how best to contact GP / contact GP for them.
